# Digital literacy in the use of digital platforms in public administration in the Lambayeque Region

**DOI:** 10.12688/f1000research.164515.1

**Published:** 2025-07-11

**Authors:** Carla Angelica Reyes Reyes, Karla Ivonne Rojas Jiménez, Onésimo Mego Núñez, Juan Amilcar Villanueva Calderón

**Affiliations:** 1 Lambayeque, Universidad Señor de Sipan, Chiclayo, Lambayeque, +51, Peru; 2 La Libertad, César Vallejo University, Trujillo, La Libertad, +51, Peru

**Keywords:** Digital Literacy, Digital Platforms, Public Administration

## Abstract

**Background:**

Nowadays, digital literacy plays a key role in improving public management, especially in the most vulnerable communities. In this regard, we aim to understand how the knowledge and use of digital tools impact the work life of the vulnerable population in Lambayeque, facilitating their access to digital platforms and, in turn, improving their interaction with public services. The research aimed to analyze how the implementation of digital literacy improved the use of digital platforms in public administration among the vulnerable working population of the Lambayeque Region during the year 2023.

**Methods:**

To achieve this, an applied approach with a descriptive-propositional scope was adopted. Additionally, a non-experimental and cross-sectional design was employed, considering a population of 1,356 inhabitants of Lambayeque, from which 673 were selected using a statistical formula.

**Results:**

The results evidenced a positive relationship between digital literacy and the use of digital platforms. It was observed that the majority of respondents had a high level of digital literacy, which favored efficiency in the use of these tools within public administration. However, it was also identified that a sector of the population still presented a medium level, highlighting the need to strengthen the development of digital skills. Furthermore, the findings coincided with previous research that emphasized the importance of digital literacy in various sectors, such as commerce, small and medium enterprises, and financial inclusion.

**Conclusions:**

Consequently, it was concluded that training in digital literacy was key to the adoption of new technologies and the utilization of digital services, thereby promoting a more active and empowered citizenship in the digital environment.

## Introduction

In an increasingly interconnected world, digital literacy is not only a technical skill but also a fundamental competency for full participation in economic, social, and civic life. Promoting digital literacy is crucial to reducing the digital divide, fostering social and economic inclusion, and preparing individuals to face the challenges and seize the opportunities of the 21st century.

The issue of digital literacy in public enterprises refers to the difficulties these entities face in training their employees in the effective use of information and communication technologies (ICT), which can limit efficiency, transparency, and the quality of the services offered. This issue is crucial because the lack of digital skills can result in ineffective administrative processes, poor information management, and a reduced capacity to innovate and respond to citizen needs. Overcoming these challenges is vital to improving public administration, optimizing resource use, and ensuring greater accessibility and security in service delivery.

Therefore, we will delve a little deeper; digital literacy in the Lambayeque region is a matter of vital importance for the development of an efficient and modern public administration. However, this region faces a series of problems that hinder the effective implementation of digital literacy. Among the main issues is the digital divide, with a significant disparity in access to the internet and technological devices between urban and rural areas. This lack of access limits educational and job opportunities for a large part of the population. Additionally, deficiencies in technological infrastructure and a shortage of quality training programs exacerbate this situation, leaving many without the necessary skills to effectively use information and communication technologies (ICT).

Cultural resistance to change is another significant obstacle, where many employees and citizens show reluctance to adopt new technologies due to a lack of familiarity and trust. Insufficient investment in digital literacy initiatives by local authorities also contributes to this issue. These problems have significant effects, such as reducing efficiency in public administration, limiting citizen participation in decision-making, and decreasing opportunities for economic and social development. Addressing these challenges is essential to ensure that Lambayeque can fully integrate ICT into its public administration, promoting a more transparent, efficient, and inclusive management.

At the International Level. In the United Kingdom, an article by Ochinanwata et al. (2024) titled “The Institutional Impact on the Digital Platform Ecosystem and Innovation” highlights that there are institutional elements that are either underdeveloped or weak to nurture an effective digital platform system, resulting in high business costs. A key cultural challenge is obtaining an honest workforce and managers. Additionally, there is a lack of effective policies, weak regulation, multiple taxes, and foreign competition, which affects local digital businesses. While cultural diversity has several merits, differences in cultural values and languages create marketing and promotion challenges. Furthermore, the low level of digital literacy among Generation Z, Millennials, and others, such as Baby Boomers and Generation X, poses a significant challenge regarding customer segmentation.

In Thailand, a study by Kraiwanit and Terdpaopong (2024) titled “Digital Transformation Model: A Study of Government Agencies in a Developing Country” reveals that the findings indicate a moderate readiness for digital technology within the OTEP, with a predominant negative attitude towards the use of data storage among employees. Technological facilities, technological literacy, leadership, and organizational culture significantly impact the utilization of digital technology. However, for young employees of the OTEP, attitude, technological literacy, and leadership do not have a significant effect on data storage consumption. This highlights the challenges of fostering human resource development, particularly in the area of digital technology, as most staff in government agencies in Thailand are of a certain age. The adoption of digital technology is essential for improving organizational performance, especially in large government agencies.

In Turkey, Tuncalp (2024), in his article titled “Shaping the Future: Integrating Artificial Intelligence in Family Businesses,” addresses the possibility that family businesses in Turkey view AI as a strategic tool to enhance operational efficiency and customer interaction. However, the integration of AI technologies often presents challenges, such as resource limitations, digital literacy gaps, and concerns about maintaining the family legacy. In particular, companies that successfully adopt AI tend to employ customized strategies that align with their core values, involving key family members in the decision-making process and fostering a culture of innovation. The study also highlights the importance of ethical considerations and governance to ensure that AI initiatives resonate with the spirit of the family business.

On the other hand, in Spain, an article by Vallès and Domènech (2024) titled “Digital Citizenship in the School: Democracy, Pragmatism, and RRI” notes that the emergence of the COVID-19 pandemic increased the use and narrative around digitalization in many areas of daily life, including education. Although many of the concerns in this regard reflect issues that have been raised previously, the use of digital technologies has intensified, and the rise of automation in many areas of life is generating new controversies and evolving in ways that are still difficult to foresee. In this context, this article argues that digital literacy is particularly relevant for our children and is a type of literacy that goes beyond the use of technological devices.

In Portugal, an article by Pires et al. (2024) titled “The Impact of Digital Burnout on the Use of Digital Consumption Platforms” highlights that as digital transition processes occur at high speed, new business models and opportunities arise. One of these phenomena is the explosion of digital consumption platforms that are changing consumption patterns as well as the boundaries of commercial exchange. The emergence of the COVID-19 pandemic triggered exponential growth of this consumption model, while simultaneously intensifying the motivation to use digital platforms. The intensive use of digital devices also threatens the mental health of digital users, exacerbating states of stress, anxiety, and digital burnout. Therefore, this study analyzes the effects of motivators and digital burnout on customer use of digital platforms in a post-pandemic context.

**Table 1.** Independent variable: Digital literacy.

		Frequency	Percentage
Valid	Low	1	0.1
	Medium	250	37.1
	High	422	62.7
	Total	673	100.0

Source: Elaboración propia.

**Table 2.** Instrumental dimension.

		Frequency	Percentage
Valid	Low	8	1.2
	Medium	251	37.3
	High	414	61.5
	Total	673	100.0

Source: Elaboración propia.

**Table 3.** Cognitive dimension.

		Frequency	Percentage
Valid	Low	1	0.1
	Medium	103	15.3
	High	569	84.5
	Total	673	100.0

Source: Elaboración propia.

**Table 4.** Emotional dimension.

		Frequency	Percentage
Valid	Low	14	2.1
	Medium	181	26.9
	High	478	71.0
	Total	673	100.0

Source: Elaboración propia.

**Table 5.** Dependent variable: Digital platforms.

		Frequency	Percentage
Valid	Low	1	0.1
	Medium	319	47.4
	High	353	52.5
	Total	673	100.0

Source: Elaboración propia.

**Table 6.** Internet access dimension.

		Frequency	Percentage
Valid	Low	5	0.7
	Medium	348	51.7
	High	320	47.5
	Total	673	100.0

Source: Elaboración propia.

**Table 7.** Programs and applications dimension.

		Frequency	Percentage
Valid	Low	1	0.1
	Medium	67	10.0
	High	605	89.9
	Total	673	100.0

Source: Elaboración propia.

**Table 8.** Operating systems dimension.

		Frequency	Percentage
Valid	Low	2	0.3
	Medium	191	28.4
	High	480	71.3
	Total	673	100.0

Source: SPSS version 27.

**Table 9.** Chi-square tests.

	Value	df	Asymptotic significance (2-sided)
Pearson Chi-Square	8.556 ^a^	4	0.073
Likelihood Ratio	9.234	4	0.056
Linear-by-Linear Association	6.996	1	0.008
N of Valid Cases	673		

Source: Elaboración propia.

**Table 10.** Symmetric measures.

		Value	Asymptotic Standard Errora	Approx. Tb ^b^	Approx. Sig.
Ordinal by Ordinal	Gamma	0.202	0.077	2.571	0.010
N of Valid Cases		673			

Source: Elaboración propia.

In Germany, an article by Bausch et al. (2024) titled “Technology-Induced Stress and Employee Resistance in the Context of Digital Transformation and Identification of Countermeasures” indicates that, in light of increasing digitalization, companies must make significant changes to their offerings and operations to remain competitive. This digital transformation of organizations includes a digital transformation of the workplace, which often faces resistance from employees. While it is recognized that reducing employee resistance is crucial for organizations, there is a limited understanding of the antecedents of employee resistance in the context of digital transformation, the different behaviors of resistance, and possible countermeasures. Based on theories of techno-stress and employee resistance, we address these research gaps. The results of two empirical studies support our central prediction that the digital transformation of the workplace causes techno-stress, which in turn promotes passive and active resistance behaviors among employees. Furthermore, we highlight that organizations can use the facilitation of digital literacy to reduce techno-stress and employee resistance.

In Ukraine, Strilchuk et al. (2024) in their work titled “Determinants of Sustainable Development in the Context of Digital Transformation” highlights that the era of digital technologies creates new approaches to solving existing problems and challenges. Therefore, life in the new normal requires the definition of global determinants of sustainable development and the creation of new tools to achieve its objectives. The study provided empirical evidence and demonstrated that information and communication technologies (ICT) are one of the important drivers of sustainable development. The analysis conducted showed that there is a direct impact of the development of ICT and digitalization on the speed of achieving SDG 9 “Industry, Innovation, and Infrastructure.”

In Singapore, a publication by Sha et al. (2024), titled “Governance of Disruptive Technologies for Inclusive Development in Cities: A Systematic Literature Review,” outlines that cities are increasingly adopting advanced technologies to address complex challenges. The application of technologies such as information and communication technology, artificial intelligence, big data analytics, and autonomous systems in the design, planning, and management of cities can lead to disruptive changes in their social, economic, and environmental composition. We expand the debate by identifying and incorporating the motivations behind the adoption of disruptive technologies and the challenges they present for inclusive development. We conclude that inclusive development in technology-driven cities can be achieved if governments develop appropriate adaptive regulatory frameworks to engage technology companies, build political capacity, and adopt more adaptive governance models. We also highlight the importance of recognizing the influence of digital literacy and smart citizenship, and exploring other dimensions of inclusivity, to govern disruptive technologies in inclusive smart cities.

In Colombia, an article by Pérez and Aleán (2024) titled “The Impact of ICT and E-commerce on Employment in Colombia” states that highly skilled workers are the ones who benefit the most from the introduction of e-commerce and its associated ICT in a company, exhibiting a high degree of complementarity compared to unskilled workers. Therefore, our findings support the hypothesis that the use of ICT and the adoption of e-commerce lead to the substitution of labor, particularly for low-skilled workers. This study recommends that policymakers invest in capacity development to meet the demands of e-commerce, focusing both on improving workers’ skills to enhance their digital literacy and on implementing specific support programs for a smooth transition to the digital economy at all qualification levels.

In Indonesia, an article written by Kamilah and Juliati (2024) titled “Optimizing Digitalization in the Face of Global Competition: The Case of Islamic Accounting” states that it is essential for Islamic higher education to integrate technology into the learning process. Adjustments must be made to meet the needs of businesses and the learning provided, contributing to an overview of the digitalization that needs to be optimized by Islamic higher education so that it can then use this framework to shape students’ competencies to prepare them for competition in the job market. This includes proposals for the digitalization of Islamic accounting through the use of accounting software, Microsoft Excel, digital literacy regarding financial technology, and AI. These proposals are important for shaping graduates’ readiness to compete in an increasingly competitive labor market.

In the present study the objective was analyze how the implementation of digital literacy improves the use of digital platforms in public administration among the vulnerable labor population of the Lambayeque region, 2023 and specific objectives were Evaluate the level of implementation of digital literacy in public administration among the vulnerable labor population of the Lambayeque region. Determine the level of use of digital platforms in public administration among the vulnerable labor population of the Lambayeque region. Describe the impact of the implementation of digital literacy on the use of digital platforms in public administration in the Lambayeque region.

Finally, the following hypothesis was proposed: The implementation of digital literacy improves the use of digital platforms in public administration among the vulnerable population of the Lambayeque region, 2023.

## Theoretical framework

International Level. In the United Kingdom, the research by Ikhwanul et al. (2023) explored the relationship between digital literacy (DL), techno-stress, and the performance of English as a Foreign Language (EFL) teachers. A correlational study with a descriptive approach was conducted, involving six teachers from different universities in East Java, Indonesia. Data collection was carried out through questionnaires, semi-structured interviews, and analysis of teaching practices. The results showed that, while teachers felt confident in their pedagogical and content knowledge, they faced difficulties integrating it with technology, which affected their performance. A strong negative correlation (-.824) was found between DL and techno-stress. In conclusion, the study underscores the importance of training teachers in the use of technology to optimize English teaching and reduce the impact of techno-stress.

In South Korea, the research by Hyunseung et al. (2024), titled “Effect of Digital Sales Preparedness on Customer-Oriented Behavior of Sellers through Digital Literacy and Self-Efficacy,” examined the impact of digital preparedness on sellers and the factors influencing their performance. In particular, the mediating role of digital literacy and self-efficacy in the relationship between digital sales preparedness and customer-oriented behavior was analyzed. Data were collected from 254 sellers at a banking and financial services company in South Korea. After excluding incomplete surveys, 150 responses were analyzed. The findings revealed that better digital preparedness strengthened digital literacy, which in turn increased self-efficacy and promoted a more customer-centered attitude. The study concluded that training in digital skills is key to optimizing sellers’ performance, as it not only enhances their confidence but also reinforces their focus on customer needs.

In Indonesia, the research by Raharjo et al. (2024), titled “Digital Literacy and Business Transformation: Sociocognitive Learning Perspectives in Small Enterprises,” analyzed how digital literacy influences the digital transformation of small businesses, considering prior factors and the role of the government in IT infrastructure. A survey was conducted with 293 small enterprises in East Java, using Partial Least Squares Structural Equation Modeling (PLS-SEM) for data analysis. The results showed that locus of control, need for achievement, and social capital significantly drove digital literacy, which in turn facilitated the digital transformation of businesses. However, IT infrastructure did not have a relevant moderating effect on this relationship. The study determined that strengthening social capital and leveraging government initiatives in business incubation and access to technology are key strategies for SMEs to face digital challenges, especially following the COVID-19 pandemic.

In South Africa, the research by Mahlatji and Mahlatji (2024), titled “Adoption of Digital Literacy for the Growth of Small Municipal Enterprises in South Africa: A Case Study of the Mankweng Municipality in Limpopo Province,” analyzed the impact of digital literacy on the growth and sustainability of small municipal businesses, with a special focus on Mankweng, Limpopo. The study examined the digital divide and its implications for business transformation and economic development. A literature review and analysis of empirical data obtained from small business owners in Mankweng were conducted. The findings indicated that, while digital literacy could be key to growth and economic inclusion, its adoption still faces cultural and structural barriers. The research concluded that South Africa requires an integrated approach that promotes digital literacy as a foundation for municipal business activity, fostering a change in attitude that drives economic participation and inclusive development.

In Hong Kong, the research by Hongyee (2024), titled “Improving Digital Literacy in Education: Educational Guidelines,” analyzed the role of digital literacy in the accumulation of online social capital and how the socioeconomic environment influences this relationship. The study, which had a quantitative approach, involved 1,747 young people aged between 13 and 30 years. For the analysis, Spearman correlations, hierarchical regression, and mediation analysis were employed. The results revealed that the creative dimension of digital literacy was the main factor in strengthening online social capital. Additionally, it was found that education had a more significant impact than demographic factors in this relationship, suggesting that proper training can compensate for socioeconomic inequalities. The study highlighted the need to implement educational programs aimed at young people, especially those in vulnerable situations, to enhance their digital skills and promote their inclusion in the digital society.

In Uganda, the research by Kiwanuka and Bongani (2024), titled “Digital Literacy, Insurtech Adoption, and Insurance Inclusion in Uganda,” explored the relationship between digital literacy, insurtech adoption, and insurance inclusion in the country. In particular, it analyzed whether insurtech adoption mediated the connection between digital literacy and access to insurance. The study, which had a correlational, quantitative, and cross-sectional approach, involved a sample of 391 individuals who had used digital platforms to access insurance services. Through Partial Least Squares Structural Equation Modeling (PLS-SEM), it was found that both digital literacy and insurtech adoption significantly influence insurance inclusion. Additionally, it was confirmed that insurtech adoption acts as a bridge between digital literacy and access to insurance. The study highlighted that, although the insurance market in Uganda remains technologically limited, strengthening digital literacy and promoting insurtech can enhance insurance inclusion in the country.

In South Korea, the research by Yeon Ha and Seongcheol (2024), titled “Development of a Conceptual Framework for Literacy in Digital Platforms,” aimed to analyze the existing literature on digital literacy and digital platforms to develop a conceptual model that evaluates literacy in these environments. The study was based on a comprehensive review of 735 scientific articles on digital literacy, which allowed for the identification of seven key constructs and three essential competencies that make up the platform literacy framework. For applicability in different contexts, the model was structured with fixed attributes and variables. The research concluded that this framework represents a significant contribution to the study of digital literacy, providing a comprehensive tool to understand and evaluate how individuals interact with digital platforms in various scenarios, which can enhance their effective use in practical environments.

In Italy, the research by Coco et al. (2024), titled “Fostering Digital Literacy in Small and Micro Enterprises: Digital Transformation as an Open and Guided Innovation Process,” analyzed how SMEs can overcome resistance to digitalization through open innovation and design thinking. The study was based on the analysis of 74 Italian SMEs that participated in an Industry 4.0 initiative. Through a nine-month action-research approach, key factors driving digitalization and compliance with policy objectives in this area were identified. The results indicated that training, networking, and co-creation play an essential role in enhancing digital literacy and technological adoption in SMEs. The study concluded that understanding both the facilitators and barriers to digitalization is crucial for designing effective strategies that help SMEs advance in their digital transformation.

In Taiwan, the research conducted by Ching (2024), titled “The Effect of Digital Literacy on Social Entrepreneurial Intentions and Emerging Behaviors Among Mass Communication Students and Professionals,” aimed to evaluate the factors influencing social entrepreneurship intentions and behaviors among mass communication students and professionals. Using a quantitative survey, information was collected from 814 participants, including 373 students and 441 professionals. The data were analyzed using structural equation modeling. The results showed direct and positive effects of perceived social support, peer social awareness, and digital literacy on social entrepreneurship intentions, as well as direct effects of these intentions on entrepreneurial behaviors. The study identified digital literacy as a key factor in fostering both intentions and behaviors related to social entrepreneurship. Additionally, educational recommendations were provided to enhance digital literacy and promote social entrepreneurship among the participants.

In Thailand, the research by Imjai et al. (2024), titled “The Interwoven Effects of Digital Literacy and Agile Mindset on Design Thinking Ability and Management Control Competence: Perspectives of Young Thai Accountants,” focused on the importance of developing digital knowledge and agility among Generation Z accountants to tackle the challenges of the digital era. The study adopted a quantitative approach, with 450 participants, primarily women (79.8%) and men (18.7%), all of whom were young accountants with university training in accounting and aged between 22 and 40 years. Through validated questionnaires and PLS-SEM analysis, it was found that both digital literacy and an agile mindset significantly influence the development of design thinking skills and management control competence. The results concluded that accountants with high levels of digital literacy and an agile mindset are more competent in applying these skills. Additionally, it was observed that design thinking acts as a partial mediator between digital literacy, agile mindset, and management control competence, suggesting areas for future research.

In India, the research conducted by Lahiri et al. (2022), titled “Digital Literacy: An Empirical Study for Fashion Design Students in India,” focused on exploring emerging variables of digital literacy, particularly competencies related to the fashion industry. Using an exploratory methodology based on literature review and a self-assessment survey, the study was applied to 120 undergraduate students from the Bachelor of Fashion Design program, who rated their digital literacy competencies on a five-point Likert scale. The results were analyzed using multivariate statistical tools. According to the competencies outlined in the UNESCO Global Framework for Digital Literacy, eight key dimensions of digital literacy were identified. ANOVA analysis showed that higher-order cognitive competencies, such as software management and digital citizenship, increased with academic progress. In contrast, lower-order competencies did not experience significant changes over time. This study may be useful for future research in similar programs within the fashion industry.

In Europe, the analysis conducted by Brînzac et al. (2024), titled “Improving Communication with Patients Through Digital Literacy of Healthcare Professionals,” focused on exploring the level of digital literacy among Romanian healthcare professionals and how this aspect could optimize communication with patients. The methodological approach adopted was qualitative, as the topic is complex and underexplored. Through 20 interviews conducted with resident and senior doctors, medical students, and nurses, the study delved into the most sensitive aspects of interaction. The interviews were recorded, transcribed, and stored in protected documents. The thematic analysis of the obtained data revealed four key themes: (a) digital health literacy, (b) communication with patients, (c) improvements for the National Health Strategy 2014-2020, and (d) time as a barrier in communication. The analysis was carried out using MAXQDA2018 software, providing in-depth insights into how to improve communication in the healthcare field through greater digital literacy.

In Spain, the study developed by Vena et al. (2024), titled “Quality of Chatbot Service: An Experiment Comparing Two Countries with Different Levels of Digital Literacy,” focused on analyzing how tourists’ digital literacy (DL) influences their perception of the quality of electronic service received. To this end, a comparative experiment was designed between two customer service technologies: email, as a traditional channel, and chatbot, as an innovative alternative. Additionally, a comparison was made between two countries with different levels of DL: Spain, with a higher level, and the Czech Republic, with a lower level. A total of 124 participants were subjected to a simulation in which they had to manage a hotel reservation through these channels. The results showed that individuals with higher DL rated the quality of service provided by the chatbot more positively, highlighting its speed and efficiency in completing the task. In contrast, participants with lower DL preferred email, perceiving it as a safer, more empathetic, and reliable option in terms of information. Finally, the research highlights that chatbots were rated better in responsiveness compared to email. These findings provide relevant information for the implementation of automated customer service systems in the tourism sector, allowing companies to tailor their services to the digital competencies of their users.

In Turkey, the research conducted by Dalgiç et al. (2024), titled “ChatGPT and Learning Outcomes in Tourism Education: The Role of Digital Literacy and Individualized Learning,” aimed to analyze the relationship between the use of ChatGPT as artificial intelligence technology and learning outcomes in tourism education. The study adopted a quantitative approach with experimental support. To ensure data accuracy, participants were asked to explore various educational topics using ChatGPT before responding to an online questionnaire. The survey was administered to tourism faculty students through Google Forms and WhatsApp groups from their universities. The findings revealed that ChatGPT has a significant impact on learning outcomes. Additionally, digital literacy plays a mediating role in this relationship, while individualized learning acts as a moderating factor. These results highlight the importance of digital literacy in the effective integration of artificial intelligence in education and suggest that personalized learning strategies can further enhance its benefits in tourism teaching.

Finally, in Taiwan, the study developed by Hsing et al. (2024), titled “The Role of Cognitive Benefits and Technology Use in Predicting Students’ Sustainable Behavior Intentions: The Moderating Roles of Data Literacy and Marketing Strategy,” analyzes the conceptual framework underlying students’ intentions and sustainable behavior. This study emphasizes the need to strengthen digital literacy among students to enhance their competitiveness in the job market. To this end, 646 students were surveyed, and structural equation modeling (SEM) was used to assess the relationship between their digital capabilities and the sustainability of their behaviors and intentions in the tourism and hospitality sectors. The results indicated that competent use of technology, such as access to high-quality websites and effective information sharing, influences both directly and indirectly the perception of sustainability, covering aspects like destination appeal and psychological impact. It was also confirmed that data literacy and marketing strategies play a moderating role in this relationship. In its final section, the study delves into the theoretical and educational implications of these findings, providing a detailed analysis of how digital literacy can promote more sustainable practices in education and the tourism industry.

The rapid evolution of digital technology has transformed the social and economic landscape globally, offering new opportunities and challenges in interacting with public services. In this context, digital literacy stands out as a crucial tool to ensure that all segments of society, especially in vulnerable labor communities with limited access to technology and digital knowledge, can take advantage of the benefits of digital platforms. This research proposes to explore how the implementation of digital literacy can significantly improve the use of these platforms in public administration in the Lambayeque region. The objective of the study is to understand and measure the impact of digital literacy on the effectiveness, accessibility, and user satisfaction when interacting with digital public services. The relevance of this research is based on various theoretical, practical, and social justifications, grounded in expert contributions and detailed analyses of existing practices.

This research will be based on the contributions of Torres (2023) and López (2022), who provide theoretical support through their concepts and theories regarding each variable involved. It is important to note that Torres contributes to the conceptualization of the digital literacy variable, as well as the definition of its dimensions; on the other hand, López provides the conceptualization of digital platforms and their respective dimensions. However, secondary authors will also be included to enrich the theoretical framework, a crucial element for a deep understanding of the problem.

The research proposes a solution to the challenges faced by the vulnerable labor population in the Lambayeque region regarding effective engagement with digital platforms in public administration, specifically addressing the lack of emphasis placed on the implementation of digital literacy. This measure will help improve the use of digital platforms through various strategies. Additionally, Ottolenghi (2022) highlights the growing importance of digital reading and writing practices in everyday interaction with online platforms. By increasing digital literacy, it is expected that members of the vulnerable labor community will not only access but also effectively engage with government digital services, which could translate into improved speed and effectiveness of public services such as administrative procedures, access to healthcare, and online educational services.

En términos sociales, la investigación propone un impacto significativo en la inclusión digital en la población laboral vulnerable de la región de Lambayeque, ya que Romero et al. (2018) argumenta que las plataformas digitales no solo facilitan transacciones comerciales, sino que también son vitales para la inclusión social y económica; es por ello que mediante el mejoramiento del alfabetismo digital, se anticipa que la comunidad laboral estará mejor equipada para utilizar estas plataformas para mejorar su acceso a oportunidades económicas, educativas y de bienestar, reduciendo la brecha digital y promoviendo la equidad social.

### Digital literacy

**Definition.** According to Dussel (2006), it is defined as “the education that allows for a critical and productive relationship with new technologies.” On the other hand, Area et al. (2012) state that digital literacy encompasses the ability to manage and understand digital technologies, enabling users to access and process information efficiently.

Additionally, Gutiérrez (2003) argues that digital literacy includes not only basic technical skills but also the critical capacity to interpret and produce information in the digital environment. Furthermore, Ontiveros et al. (2006) operationally define digital literacy as the ability to perform specific tasks in digital environments, such as document editing and secure interaction on social networks.

### Characteristics

Gutiérrez (2003) describes the characteristics of digital literacy, highlighting the ability to use information in various formats from digital sources.

### Dimensions

Torres (2023) emphasizes that the dimensions encompassing digital literacy are:

- Instrumental
- Cognitive
- Emotional

Nevertheless, within our research, we will consider only those dimensions that are necessary for the application of our instrument.

### Digital platforms

**Definition.** Fabián (2020) defines digital platforms as technological structures that facilitate interactions between different users, providing access to resources and services.

From a different perspective, Ottolenghi (2022) explains that digital platforms serve as intermediaries between various user groups, such as consumers and producers. Meanwhile, Romero et al. (2018) operationally describe digital platforms in terms of their usability, diversity of services, and technological integration capacity.

### Objective

According to Fabián (2020), the main objective of digital platforms is to provide an effective means for the distribution of products and services on a global scale.

### Characteristics

Ottolenghi (2022) highlights that a key characteristic of digital platforms is their ability to scale rapidly, adapting to a large number of users and transactions.

### Types

Romero et al. (2018) identify several types of digital platforms, including those dedicated to e-commerce, online education, and social networks.

### Dimensions

According to López’s (2022) observations, three crucial aspects are highlighted to determine the dimensions based on the implementation of digital platforms, which are as follows:

- Internet access
- Programs and applications
- Operating systems

## Methods

### Type, design, and scope

The present research falls under a specific type based on its scope, reach, and focus. Therefore, this exploration is categorized within the realm of applied research, as described by Hernández et al. (2014), since its purpose is to understand the “how to do” and to act, meaning to modify, maintain, reform, or radically change some aspect of social reality. Consequently, the research aims to achieve new knowledge that allows for solutions to practical problems.

Based on the analysis conducted, it was determined that the research was focused on a quantitative approach, according to Hernández et al. (2014). This type of study allowed for the breakdown of the research object into its various elements, facilitating a detailed analysis and helping to accurately address the stated objectives. Additionally, it provided the opportunity to make well-founded generalizations based on the results obtained.

The design of our research is non-experimental and cross-sectional. According to Maxwell (2019), in a non-experimental design, data is collected without manipulating the study variables, which is crucial when evaluating interventions in real contexts where it is not possible or ethical to directly manipulate the participants’ conditions. This design allows for the observation of the natural conditions in which digital literacy develops and its impact on the use of digital platforms.

Additionally, the research has a descriptive-propositional scope. Ramos (2021) defines this approach as one that not only describes the current characteristics and conditions of a phenomenon but also suggests measures based on the observed findings. This approach is fundamental for understanding the current conditions of digital literacy in the community and proposing effective improvements in the use of digital platforms.

On the other hand, the cross-sectional nature of the research, as described by Ramos (2021), implies that data is collected at a single point in time. This is especially useful for obtaining a snapshot of current conditions and evaluating the effectiveness of public policies and educational programs at a specific moment, facilitating comparative analysis with other communities or time periods.

### Population and sample

According to the definition by Otzen and Manterola (2017), the population in a research study is defined as the total set of individuals who share specific characteristics relevant to the study, from which a representative sample will be drawn.

Similarly, Condori (2020) describes the study population as the complete group of subjects or elements that possess the properties the researcher wishes to study, and on which the results of the research are intended to be generalized.

Therefore, in our study, a population of 1,356 inhabitants from Lambayeque was considered as the focus of evaluation.

Likewise, Gregorio (2023) tells us that a probabilistic sample is one that aims to estimate the probability that each unit of analysis belongs to the sample. It can be noted that there are simple random sampling, systematic random sampling, stratified sampling, and cluster sampling. Therefore, our study emphasizes the use of stratified sampling; as Mucha et al. (2021) describe stratified probabilistic sampling as a technique that divides the population into homogeneous subgroups before random selection, ensuring that the sample is representative of the population as a whole and increasing the statistical precision of the results.

In light of this, by applying the formula and dividing the subgroups, we were able to obtain a sample according to the statistical formula of a total of 673 inhabitants from Lambayeque, which will be of great help for this research.

In this research, a vulnerable working population was considered, and among these selection criteria, priority was given to older adults, as they provide the most identification in relation to this digital adaptation.

### Procedure

The procedure for conducting this study is divided into several key stages that allow us to understand the phenomenon in question within its context. It begins with a general idea, followed by the formulation of the problem to be addressed. Then, we design the methodology, select the appropriate samples, collect and process the information, and finally, interpret and analyze the results obtained to draw meaningful conclusions.

### Techniques and instruments for data collection

According to Mendoza and Avila (2020), techniques and instruments for data collection are crucial for acquiring relevant and precise information, using methods that range from structured surveys to in-depth interviews and direct observation.

For this study, the survey was used as the main technique for information collection. According to Santisteban (2014), this method involved formulating questions to a specific group with the purpose of obtaining relevant data on a research topic. This technique was chosen because it allowed for the design of strategic questions that facilitated the analysis of the identified problem and contributed to the acquisition of key information for the study.

Additionally, a questionnaire was used as a tool to collect data in a more detailed manner. As explained by Santisteban (2014), this tool allowed the researcher to formulate a series of questions aimed at obtaining precise information from a sample of people. In this case, the questionnaire consisted of 20 questions, distributed across three dimensions for each study variable. It was applied to the previously selected sample in order to obtain results that would validate the research hypothesis. To measure the responses, a Likert scale was used, ranging from “strongly disagree” (1) to “strongly agree” (5), which allowed for a structured analysis of the participants’ perceptions.

Regarding the observation about the evaluation tool used, it is stated that the research instrument is of original authorship. Therefore, obtaining a third-party copyright license is not applicable.

### Validity and reliability

According to Sánchez (2022), the validity of data collection instruments ensures that they effectively measure what they are designed to measure, which is essential for obtaining legitimate and applicable results. On the other hand, the validity of this study was supported by experts who were licensed in public administration and specialized in human resources to better understand the relationship of digital adaptability in public digital platforms.

Similarly, Mendoza and Avila (2020) emphasize reliability as the consistency of a measurement instrument, indicating that an instrument is reliable if it produces stable and coherent results under consistent conditions over time. Therefore, the reliability of this research was assessed using the statistical software SPSS V.28. This program, through Cronbach’s Alpha, allows us to establish the reliability of the questionnaire. The closer the value is to one, the greater the reliability of the instrument; in our case, the reliability was found to be 0.841 with a total of 20 items.

### Data analysis

On the other hand, Torre and González (2020) describe data analysis procedures as the application of statistical methods and qualitative techniques to interpret and transform the collected data into meaningful information that can be used to answer research questions, support or refute hypotheses, and formulate recommendations.

To carry out the analysis of the information, it was first necessary to convert the obtained data into manageable formats that facilitated its interpretation. The study focused on understanding the situation of the vulnerable working population in the Lambayeque region and its relationship with digital platforms of public administration. To this end, clear and precise objectives were defined, which allowed for a systematic and effective procedure, ensuring that the conclusions were well-founded and useful for the study.

To collect the information, a sample of 673 inhabitants from Lambayeque was surveyed. During this process, each obtained data point was carefully reviewed to detect possible errors and ensure the accuracy of the results. This step was crucial to ensure the reliability of the information and to obtain a solid analysis of the identified problem.

The questionnaire used was based on the Likert scale, which allowed for a structured measurement of the respondents’ perceptions. Once the responses were collected through a digital form, the data were organized in Excel and numerically coded. Then, they were processed using IBM SPSS Statistics V.28, employing Cronbach’s alpha coefficient to assess the reliability of the information. Finally, the results were formatted according to APA guidelines, ensuring that each statistical analysis was correctly interpreted and could provide valuable information for the study.

### Ethical criterial

During the research process, 673 individuals were selected and provided their informed consent, allowing us to obtain the data necessary for the study. This consent was obtained following the principles of intellectual honesty, transparency, respect for intellectual property, and accountability. The study was approved by Board of Directors Resolution No. 121-2023/PD-USS.

This study has been approved by the Institutional Research Ethics Committee of the Universidad Señor de Sipán, in accordance with the USS Code of Research Ethics (code 0455-10102023-CIEI, dated 10-10-2023). The approval number assigned to this study is BOARD OF DIRECTORS RESOLUTION No. 053-2023/PD-USS.

The research was conducted in line with the principles established in the Declaration of Helsinki on research involving human subjects. All participants were informed about the study’s objectives and gave their consent before participating. Data confidentiality, the right to privacy, and the right to withdraw at any time without consequences were guaranteed. For minor participants, informed consent was obtained from both their parents or legal guardians and the minor, in accordance with the current Clinical Trials Regulations of the National Institute of Health (INS, Peru).

The study does not involve medical interventions or experimental procedures that jeopardize the integrity of the participants. Likewise, the standards for the protection and well-being of research subjects will be respected, in accordance with institutional guidelines and applicable national and international regulations.

### Informed consent

In the present study titled *Digital Literacy in the Use of Digital Platforms in Public Administration in the Lambayeque Region*, a sample of 673 inhabitants from the Lambayeque region was used. This sample was determined through the application of an appropriate statistical formula and the corresponding division into subgroups, thus allowing for an accurate representation of the target population. It is important to note that all participants provided written informed consent, which was requested and collected prior to the administration of the research instruments, ensuring compliance with ethical principles and the confidentiality of the information provided, as detailed in the methods section of this article.

### Duration of the study

In the Methods section, we requested the inclusion of the specific dates for the duration of the study. The data for this research were collected over a two-month period, specifically in January and February 2025. These months were selected to ensure that the sample would be representative and to align with the availability of participants. During this time, all the necessary instruments and procedures were applied to gather accurate and reliable data. This period also allowed for sufficient time to ensure the informed consent of all participants was obtained and recorded before the commencement of data collection.

## Results

Note: The results of the table and figure reflect that the majority of the surveyed individuals have a high level of digital literacy, as more than half, specifically 62.7%, easily master these skills. On the other hand, 37.1% are at an intermediate level, indicating that while they have knowledge in the area, they can still strengthen their capabilities. In contrast, only 0.1% present a low level, suggesting that the digital divide within this group is minimal. Overall, these data demonstrate that the vast majority of participants are well-prepared to navigate digital environments, although there remains a sector that could benefit from greater opportunities for learning and development in this area.

Note: In general terms, observing the table and figure, the results show that a large portion of the respondents, at 61.5%, possess a high level in the instrumental dimension, suggesting a good ability to handle digital tools in various contexts. Additionally, 37.3% are at an intermediate level, indicating that while they have knowledge in the area, they could still strengthen certain skills to optimize their performance. On the other hand, only 1.2% present a low level, demonstrating that the number of people with difficulties in this dimension is minimal. These data reflect a positive outlook regarding the mastery of digital tools, although they also highlight the importance of continuing to promote training opportunities for those still at intermediate or low levels.

Note: Evidently, the results reveal that a large percentage of the respondents, specifically 84.5%, possess a high level in the cognitive dimension, indicating a strong ability to process, understand, and apply information in digital environments. Additionally, 15.3% are at an intermediate level, suggesting that while they have cognitive skills in this area, they can still develop more critical and strategic thinking in the use of technology. On the other hand, only 0.1% present a low level, demonstrating that difficulties in this dimension are practically nonexistent within the sample. Overall, these results reflect a promising outlook regarding the development of digital cognitive skills, although they also highlight the need to continue strengthening the competencies of those still at intermediate levels.

Note: According to the observed table and figure, the results show that the majority of respondents, at 71.0%, present a high level in the emotional dimension, indicating that they have good management of their emotions in the use of digital environments, maintaining a balanced and resilient attitude in the face of technological challenges. Additionally, 26.9% are at an intermediate level, suggesting that while they have some emotional control in the digital realm, they can still improve in areas such as stress regulation or confidence in handling technological tools. On the other hand, 2.1% show a low level, indicating that there is a small group that may experience greater emotional difficulties in this context. Overall, these data reflect a positive trend, where the majority of participants manage to navigate emotionally well in the digital world, although the need to provide support to those still facing challenges in this area persists.

Note: Observing the table and figure, the results reflect a relative balance in the use of digital platforms, as 52.5% of respondents present a high level, indicating considerable mastery in interacting with, managing, and leveraging these environments. However, 47.4% are at an intermediate level, suggesting that while they use digital platforms with some frequency, they can still optimize their handling to enhance their experience and productivity. In contrast, only 0.1% show a low level, which indicates that difficulties in this area are minimal within the sample. Overall, these results demonstrate active participation in digital environments, although they also highlight the importance of continuing to promote strategies that strengthen the efficient and critical use of these tools among those who have not yet achieved advanced mastery.

Note: Clearly, as observed in the table and figure, the results show an almost equitable distribution in internet access, with 51.7% of respondents rating at an intermediate level and 47.5% at a high level. This suggests that while a significant portion of the sample has constant and adequate access to the network, there is still a similar proportion facing certain limitations, whether in terms of quality, stability, or availability of the service. On the other hand, only 0.7% present a low level, indicating that cases of restricted access are few but not nonexistent. Overall, these results demonstrate that while the majority of participants have sufficient connectivity, there is still a need to work on improving infrastructure and access conditions to ensure that more people can enjoy a smooth and uninterrupted digital experience.

Note: According to the observed results, it is evident that the vast majority of respondents, at 89.9%, rated at a high level in the use of programs and applications, indicating solid and frequent handling of these tools in various contexts. On the other hand, 10.0% are at an intermediate level, suggesting that while they have knowledge and experience in this area, they can still improve in the advanced use of certain functions or in exploring new digital tools. In contrast, only 0.1% present a low level, demonstrating that difficulties in this dimension are practically nonexistent within the sample. Overall, these results reflect a very positive outlook, where the majority of participants navigate the use of software and applications with ease, although there remains a small proportion that could benefit from training strategies to optimize their digital skills.

Note: As observed in the table and figure, the vast majority of respondents, at 89.9%, possess a high level in the use of programs and applications, indicating solid and frequent handling of these tools in various contexts. On the other hand, 10.0% are at an intermediate level, suggesting that while they have knowledge and experience in this area, they can still improve in the advanced use of certain functions or in exploring new digital tools. In contrast, only 0.1% present a low level, demonstrating that difficulties in this dimension are practically nonexistent within the sample. Overall, these results reflect a very positive outlook, where the majority of participants navigate the use of software and applications with ease, although there remains a small proportion that could benefit from training strategies to optimize their digital skills.

Note: According to a general analysis of the three tables, the results suggest that digital literacy does have an impact on the use of digital platforms, although with a moderate relationship. However, the Pearson chi-square test presents an asymptotic significance value of 0.073, which is close to but above the conventional threshold of 0.05, implying that it cannot be stated with certainty that the relationship between the two variables is statistically significant. Nevertheless, the linear-by-linear association test shows a clearer relationship, with a significance of 0.008, suggesting that there is a consistent trend where higher digital literacy correlates with greater use of digital platforms. Additionally, the Gamma coefficient of 0.202 with a significance of 0.010 indicates that the relationship is moderate but significant.

In summary, although the impact is not completely strong or decisive, the results indicate that a higher level of digital literacy does facilitate and promote more frequent and competent use of digital platforms. This suggests that, while other factors also influence, the level of digital literacy plays a relevant role in how individuals interact with digital technologies.

## Discussion - Conclusions - Recommendations

According to the results obtained and emphasizing the general objective regarding the analysis of how the implementation of digital literacy improves the use of digital platforms among the vulnerable working population in the Lambayeque Region, a clear and positive relationship between both factors is demonstrated. First, it was observed that the majority of respondents rated their digital literacy level as high, which contributes to the efficient use of digital platforms in public administration. However, it was also evident that a percentage of the population is still at a medium level, suggesting the need to continue promoting the development of digital skills in this area to further improve interaction with the platforms. This finding connects with previous research, such as that of Hyunseung et al. (2024), which found that digital readiness and digital literacy play a fundamental role in enhancing customer-oriented behavior among vendors, suggesting that greater mastery of digital platforms leads to more effective interaction with them. On the other hand, the study by Coco et al. (2024) highlights the importance of training initiatives in digital literacy within small and medium enterprises, reinforcing the idea that strengthening these competencies is key to adopting new technologies. Additionally, the work of Kiwanuka and Bongani (2024) in Uganda underscores that both digital literacy and the adoption of technological platforms significantly influence insurance inclusion, emphasizing how mastery of digital tools affects interaction with online services. Finally, Yeon Ha and Seongcheol (2024) argue that robust digital literacy is essential for active and productive participation in digital platforms, as observed in their conceptual framework for literacy in digital platforms. These results support the idea that the implementation of digital literacy has a direct impact on the use of digital platforms, improving interaction with public administration technologies. Torres (2023) and López (2022) reinforce this perspective by stating that a higher level of digital literacy enables users to make more efficient use of digital platforms, optimizing interaction with online services and fostering the development of a more active and digitally empowered citizenship.

Continuing with the results obtained in the first specific objective regarding the level of implementation of digital literacy in public administration for the vulnerable working population of the Lambayeque region, a high level of digital preparedness is shown in this group. More than half of the respondents (62.7%) indicated having a high level of digital literacy, reflecting a solid capacity to handle and understand digital technologies. However, 37.1% are at a medium level, suggesting that while they possess sufficient knowledge, they can still improve their digital skills. This scenario aligns with findings by Ching (2024), who noted that digital literacy plays a key role in fostering social entrepreneurship intentions, demonstrating that those with greater digital mastery have better development opportunities. Similarly, the research by Hyunseung et al. (2024) in Korea reaffirms that digital literacy is essential for improving customer-oriented behavior, suggesting that even in public administration, employees with better digital competencies have a greater capacity to interact with citizens. On the other hand, the study by Kiwanuka and Bongani (2024) in Uganda found that digital literacy also mediates access to services, such as insurance, highlighting the importance of digital literacy in inclusion and the provision of public services. Additionally, the research by Raharjo et al. (2024) on small businesses in Indonesia showed that strong digital literacy can drive transformation and organizational growth, reinforcing the idea that adequate digital literacy is fundamental for efficiency in the workplace and administration. According to Area et al. (2012), digital literacy not only encompasses the ability to use technologies but also involves the capacity to access, understand, and process information effectively, which is reflected in the results of this study, where the majority of respondents are well-prepared, although there is still a sector with potential for improvement.

Continuing with the specific objective of determining the level of use of digital platforms in public administration for the vulnerable working population in the Lambayeque region, a balanced picture is shown. A total of 52.5% of respondents rated their level of use as high, indicating a considerable capacity to interact with and effectively manage digital platforms. However, 47.4% are at a medium level, suggesting that while they are becoming familiar with these tools, they can still optimize their usage to maximize the benefits they offer. This finding is consistent with studies by Coco et al. (2024), who state that microenterprises must overcome resistance to digitalization in order to integrate technologies efficiently. On the other hand, the analysis by Hongyee (2024) highlights that, in educational contexts, digital literacy is fundamental for improving participation in online platforms, reflecting the importance of a critical and well-managed use of digital tools. Similarly, Kiwanuka and Bongani (2024) found that the adoption of digital technologies enhances inclusion in services, such as insurance, emphasizing how the use of digital platforms can facilitate the provision of public services. Additionally, the work of Yeon Ha and Seongcheol (2024) regarding the framework for literacy in digital platforms underscores the need to build adequate competencies for interaction in digital environments, while also highlighting that there are still areas for improvement in the management and utilization of these platforms. According to Ottolenghi (2022), digital platforms act as intermediaries between various user groups, efficiently connecting consumers with producers, which in the context of public administration implies that mastery of these platforms is key to improving interaction between officials and citizens.

Finally, according to the results obtained regarding the impact of digital literacy implementation on the use of digital platforms in public administration in the Lambayeque Region, there is evidence of a positive impact, although not completely determinative, between both factors. As the level of digital literacy among respondents increases, so does the competent and frequent use of digital platforms, indicating that mastery of digital tools enhances interaction and utilization of these platforms. This finding aligns with the research by Raharjo et al. (2024), who found that higher digital literacy has a significant impact on the digital transformation of small businesses, suggesting that competence in using digital technologies facilitates the adaptation of technological platforms. Similarly, Kiwanuka and Bongani (2024) demonstrated that digital literacy positively influences the adoption of technological platforms, highlighting the importance of developing these competencies to improve inclusion and interaction with digital services. In the educational field, the study by Hongyee (2024) also showed that the development of digital literacy enhances the accumulation of online social capital, reflecting how mastery of digital platforms impacts individuals’ ability to interact and benefit from them. Lastly, Yeon Ha and Seongcheol (2024) pointed out that digital literacy is an essential pillar for successful interaction with technological platforms, evidencing that adequate preparation in digital literacy facilitates the efficient use of these platforms. In conclusion, these results support the idea that digital literacy is a key factor for improving the use of digital platforms, as indicated by Torres (2023) and López (2022), who affirm that a higher level of digital literacy empowers users to better leverage technological tools, facilitating interaction and enhancing the experience in digital environments.

According to the general objective, it was concluded that the implementation of digital literacy had a positive impact on the use of digital platforms among the vulnerable working population in the Lambayeque Region. The results showed that a high level of digital literacy favored the efficient use of these tools in public administration, while a segment of the population was still at a medium level, highlighting the need to continue strengthening these competencies. Furthermore, it was confirmed that these findings coincided with similar research, which emphasized the importance of digital literacy in improving interaction with technological platforms and its influence in various sectors, such as commerce, small and medium enterprises, and insurance inclusion. In this sense, it was confirmed that training in digital literacy was key to the adoption of new technologies and the utilization of digital services, thus promoting a more active and empowered citizenship in the digital environment.

Continuing with the first specific objective, it was concluded that the implementation of digital literacy in public administration for the vulnerable working population in the Lambayeque Region reflected significant digital preparedness in this group. It was observed that a considerable portion of respondents demonstrated a high level of digital literacy, indicating a solid capacity to handle and understand digital technologies. However, there was still a sector with an intermediate level, which evidenced the need to continue strengthening their digital skills. This corroborated that overcoming resistance to digitalization and promoting participation in online platforms in various areas, such as education and administration, was key to improving interaction with digital services. Additionally, it was reaffirmed that digital platforms play a fundamental role as intermediaries in public administration, facilitating communication between officials and citizens, making their mastery essential for more efficient and accessible management.

Continuing with the second specific objective, it was concluded that the use of digital platforms in public administration among the vulnerable working population in the Lambayeque Region showed a balance between those who managed them efficiently and those who still faced certain difficulties. While a significant portion of respondents demonstrated a good level of mastery, another group still exhibited an intermediate handling, which evidenced the need to continue strengthening their digital competencies to fully leverage these tools. In this regard, the findings highlighted that digitalization in educational environments enhances participation in online platforms and that the adoption of digital technologies facilitates access to various services. Additionally, it was reaffirmed that digital platforms served as a key bridge between officials and citizens in public administration, making a stronger mastery essential to optimize management and interaction in this environment.

Finally, according to the results obtained in the third specific objective, it was concluded that the implementation of digital literacy had a positive impact on the use of digital platforms within public administration in the Lambayeque Region, although this was not completely determinative. It was observed that as individuals acquired greater digital skills, their interaction with these tools became more efficient and frequent, confirming the importance of technological mastery to optimize their utilization. Additionally, it was reaffirmed that solid digital preparation allowed users to navigate technological environments with greater autonomy and confidence, thereby promoting a more enriching and efficient experience in the use of digital platforms.

### Ethics and consent

In the research process, 673 individuals selected from the population participated. Participation began with the sending of an email to each individual, requesting their informed consent, which was received in writing through the same medium. Additionally, each participant was provided with a detailed explanation of the study, its objectives, and the methodology to be followed. Various digital tools were used for data collection, such as live meetings on Zoom and surveys via Google Forms.

The study was conducted in accordance with the ethical principles of the Declaration of Helsinki, which safeguard individuals involved in research. Each participant was clearly informed about the study’s objectives and gave their free and voluntary consent before participating. Personal information was carefully kept confidential, each person’s privacy was respected, and participants were allowed to withdraw from the study at any time without any consequences.

All participants provided their informed consent before beginning their participation. They were given a written form that described the study’s objectives, procedures, benefits, potential risks, and confidentiality conditions. The participants signed this document before completing the questionnaire. The study was conducted in accordance with the approval granted by the Institutional Ethics Committee in Research of the Universidad Señor de Sipán, with code 0455-10102023-CIEI, dated 10-10-2023. The approval number assigned to this study is BOARD OF DIRECTORS RESOLUTION No. 053-2023/PD-USS, adhering to the ethical principles established in the Declaration of Helsinki and the current national regulations.

The ethics committee did not grant any exemptions to the consent process, as the study’s methodology required data collection via a questionnaire. It was considered essential to ensure that each participant provided explicit and formal consent.

Furthermore, participants were guaranteed the right to withdraw at any time without negative consequences, and the confidentiality of the information was ensured by using coded responses to prevent any possibility of personal identification.

### Informed consent

Data are available under the terms of the Creative Commons Attribution 4.0 International license (CC-BY 4.0).

### Reporting guidelines

Zenodo. Digital literacy in the use of digital platforms in public administration in the Lambayeque Region. https://doi.org/10.5281/zenodo.15641137 ( Villanueva et al., 2022).

## Data Availability

Zenodo. Digital literacy in the use of digital platforms in public administration in the Lambayeque Region. https://doi.org/10.5281/zenodo.15641137 ( Villanueva et al., 2022). This article contains the following extended data:
• Code of Ethics • Database • F1000 Data • CIEI approval report Data are available under the terms of the Creative Commons Attribution 4.0 International license (CC-BY 4.0).
