# Supplementary material for: Digital literacy in the use of digital platforms in public administration in the Lambayeque Region
Source: F1000Res. 2025 Jul 11;14:688. [Version 1] doi: 10.12688/f1000research.164515.1 (PMC12595294; doi:10.12688/f1000research.164515.1)
Supplement: Peer review history

# Reviewer report: Reviewer response for version 1

**DOI:** 10.5256/f1000research.181040.r417000

**Published:** 2025-11-07

**Authors:** Frederico Branco

**Affiliations:** 1 University of Trás-os-Montes e Alto Douro, Vila Real, Portugal

The study examines whether digital literacy among a vulnerable working population in Peru’s Lambayeque Region is associated with the use of digital platforms in public administration. It employs a non-experimental, cross-sectional survey of 673 residents, using a 20-item Likert questionnaire with reported internal consistency, and analyzes association through contingency tests.

 The topic is timely and relevant for public service delivery, and the paper benefits from a large sample, stated reliability, clear ethics and consent, and open data availability. At the same time, methodological reporting is incomplete, and parts of the results are overly descriptive; some conclusions overreach the scope of the cross-sectional evidence. With focused revisions to tighten the reporting, align claims with statistics, and condense the narrative, the manuscript could make a valuable regional contribution.

 The conclusions should be rephrased to align with the evidence. The contingency analysis yields a non-significant Pearson χ² (p=0.073) but a significant linear-by-linear trend (p=0.008) and a modest Gamma (0.202, p=0.010). Claims that digital literacy training was “key” and that implementation has a “direct impact” exceed what these cross-sectional, primarily descriptive findings support. Reword conclusions to emphasize association rather than causation, and specify which test underpins the inference.

 Key methodological details need to be reported for reproducibility. Describe the sampling strategy beyond “statistical formula,” including any stratification, the rationale for prioritizing older adults, inclusion/exclusion criteria, recruitment, response rate, and handling of missing data. Please provide the complete questionnaire (or a stable link) and clarify whether the items form subscales used in the analysis. State the statistical analysis plan a priori (primary vs secondary tests), report effect sizes with confidence intervals, and reconcile the software version inconsistency (SPSS v28 mentioned in reliability vs “SPSS version 27” in a table).

 The results section should be condensed and analytically focused. Much of the text restates table percentages in narrative form. Replace repetition with concise synthesis that addresses the research objective and interprets the magnitude and direction of the associations. Where multiple tests are presented, justify their use and hierarchy.

 The aims and motivation are clear, but the discussion leans heavily on international examples at the expense of local policy implications. Tighten this section by explicitly connecting findings to Lambayeque’s administrative context and by distinguishing what is novel in this setting relative to prior work.

 Instrument validity is described as expert-judged, and reliability as α=0.841, which is acceptable. To strengthen credibility, specify the number and expertise of validators, any content validity indices used, and whether reliability was re-estimated by subscale. Clarify whether any pretesting or cognitive interviewing informed item wording.

 The aims, rationale, and objectives are explained clearly in the abstract and methods; retain this clarity in the discussion by foregrounding the study’s contribution to local policy decisions rather than relying on distant analogies.

 The statistical analysis is directionally appropriate; however, the hierarchy of tests and effect reporting requires clarification. Identify the primary test, provide confidence intervals for Gamma, and, if proportional-odds or ordered-logit models are feasible with your data, consider them as a robustness check to summarize the trend with covariate adjustment; otherwise, justify the chosen non-parametric approach.

Is the work clearly and accurately presented and does it cite the current literature?

Partly

If applicable, is the statistical analysis and its interpretation appropriate?

Partly

Are all the source data underlying the results available to ensure full reproducibility?

Yes

Is the study design appropriate and is the work technically sound?

Partly

Are the conclusions drawn adequately supported by the results?

Partly

Are sufficient details of methods and analysis provided to allow replication by others?

Partly

Reviewer Expertise:

Information Systems; Digital Transformation; Technology Adoption

I confirm that I have read this submission and believe that I have an appropriate level of expertise to confirm that it is of an acceptable scientific standard, however I have significant reservations, as outlined above.

# Reviewer report: Reviewer response for version 1

**DOI:** 10.5256/f1000research.181040.r416995

**Published:** 2025-09-29

**Authors:** Lorena Mihelac

**Affiliations:** 1 Information Studies in Novo mesto, Novo Mesto, Slovenia; 2 IT, Solski center Novo Mesto (Ringgold ID: 187330), Novo mesto, Novo mesto, Slovenia

Article Title: *Digital literacy in the use of digital platforms in public administration in the Lambayeque Region*

 Summary of the Article:

 The article examines the correlation between digital literacy and the utilization of digital platforms in public administration among at-risk communities in Lambayeque, Peru.

 The findings of a cross-sectional survey of 673 participants show that a more frequent and efficient platform use correlates with higher digital literacy, despite a considerable number of participants having basic digital skills. The article further outlines the importance of establishing sufficient digital training to make public services more efficient and open to everyone.

 This article significantly enhances the examination of digital literacy in the realm of public administration. It gives us a unique look at how digital skills affect access to and interaction with public services by looking at the Lambayeque Region and focusing on vulnerable groups. The methodology is clearly explained, the ethical issues are thoroughly considered, and the results show that there is a beneficial link between digital literacy and the effective use of digital platforms.

 The explanation may be improved by presenting the statistical results more clearly, especially when it comes to the moderate relationships that were found. Moreover, the conclusions might be enhanced by a more detailed examination of local policy ramifications, including efforts to mitigate gaps between urban and rural people and to assist groups with intermediate digital competencies. Lastly, a more explicit recognition of the study's limitations—like the use of self-reported data and the cross-sectional design—would assist in putting the results in the right context.

 In general, the article adds to our understanding of an important topic, and by improving some parts, it can give both academic and practical information about how digital literacy might enhance the delivery of public services.

 1. Is the work clearly and accurately presented, and does it cite the current literature?

 Partly. The article (in general) is well written. It has a clear structure, tables, and detailed explanations. It cites a broad and up-to-date range of international literature, which is a strength. However, some sections are lengthy and repetitive, and the connection between global studies and the Lambayeque context could be emphasized more directly.

 More detailed:

1. Introduction (pp. 2–4)
  - The section reviews many international studies (UK, Thailand, Turkey, Spain, Portugal, Germany, Ukraine, Singapore, Colombia, Indonesia, etc.).
  - This displays depth, but the high number of references makes it tougher to see the straight link to Lambayeque. Several of these studies could be summarized more concisely, focusing only on those most relevant to public administration and digital literacy.
2. Theoretical Framework (pp. 5–7)
  - The article again lists multiple international studies (South Korea, Indonesia, South Africa, Hong Kong, Uganda, Italy, Taiwan, India, etc.).
  - There is some overlap with the literature already discussed in the introduction, leading to repetition. These could be integrated into one streamlined section or presented as a comparative synthesis rather than individual summaries.
3. Discussion (pp. 14–16)
  - The discussion frequently cites studies already mentioned in the introduction and theoretical framework (e.g., Hyunseung et al., Coco et al., Kiwanuka & Bongani, Yeon Ha & Seongcheol).
  - While it is important to connect findings to prior research, the repetition of citations without new synthesis makes the section longer than necessary.

In short: The Introduction and Theoretical Framework may be improved by eliminating redundant international cases, and the Discussion might be more succinct by concentrating on the contributions of the current study instead of reiterating previous material.

 2. Is the study design appropriate, and is the work technically sound?

 Yes. The cross-sectional, non-experimental design is suitable for the specified purposes. The sampling method (stratified) and the reliability test (Cronbach's alpha) show that the work is technically sound.

 3. Are sufficient details of methods and analysis provided to allow replication by others?

 Yes. The technique is thoroughly explained, encompassing sample selection, questionnaire formulation, ethical approvals, and data processing protocols. Other researchers would be able to repeat these studies based on these descriptions.

 4. If applicable, is the statistical analysis and its interpretation appropriate?

 Yes.The use of chi-square, gamma coefficient, and reliability analysis is appropriate. However, interpretation could be improved: the chi-square test was not statistically significant, which should be discussed more clearly, as it affects the strength of the conclusions.

 5. Is the study of sufficient novelty and interest for publication?

 Yes **.** The article provides access to extended data (database, ethics documents, etc.) via Zenodo, ensuring transparency and reproducibility.

 6. Are the conclusions drawn adequately supported by the results?

 Partly. The findings corroborate the overarching conclusion that digital literacy enhances platform utilization. The moderate connection and non-significant chi-square test indicate that conclusions should be formulated with greater caution.

Is the work clearly and accurately presented and does it cite the current literature?

Partly

If applicable, is the statistical analysis and its interpretation appropriate?

Yes

Are all the source data underlying the results available to ensure full reproducibility?

Yes

Is the study design appropriate and is the work technically sound?

Yes

Are the conclusions drawn adequately supported by the results?

Partly

Are sufficient details of methods and analysis provided to allow replication by others?

Yes

Reviewer Expertise:

Informatics, e-platforms and cognitive gains, gamification, perception of music

I confirm that I have read this submission and believe that I have an appropriate level of expertise to confirm that it is of an acceptable scientific standard.

# Reviewer report: Reviewer response for version 1

**DOI:** 10.5256/f1000research.181040.r416992

**Published:** 2025-09-29

**Authors:** Lorena C. Espina-Romero

**Affiliations:** 1 Universidad San Ignacio de Loyola, Lima District, Lima Region, Peru

Peer Review Report

 Introductory Paragraph

 This report provides a critical evaluation of the study *“Digital literacy in the use of digital platforms in public administration in the Lambayeque Region.”* The analysis identifies the main weaknesses in each section of the manuscript and offers concrete suggestions for improvement, supported by clear examples. The goal is to help the authors strengthen the clarity, methodological rigor, and regional relevance of their work, ensuring a stronger academic contribution to the field of digital literacy and public administration.

 Abstract:

- Weakness: The abstract is too long and mixes justification, objectives, and results without hierarchy.
- How to improve: Reduce it to a maximum of 250 words, highlighting the objective, method, main findings, and conclusion.
- Example of improvement:
*“This study analyzes the impact of digital literacy on the use of digital platforms in public administration in Lambayeque, Peru. A cross-sectional survey of 673 participants showed that higher levels of digital literacy are associated with more effective use of public digital services. The results show progress but also reveal gaps in connectivity and skills. The findings support the need for targeted training programs to foster a more inclusive and efficient public administration”.*

Introduction:

- Weakness: Too many international examples dilute the focus on Lambayeque.
- How to improve: Keep the global references but explicitly connect them to the local problem.
- Example of improvement:
*“While international studies show that digital literacy improves efficiency in public institutions (Kraiwanit & Terdpaopong, 2024), in Lambayeque these challenges are intensified by the digital divide between rural and urban areas”.*

Theoretical Framework:

- Weakness: Multiple definitions are presented but not integrated into a comparative framework.
- How to improve: Synthesize definitions and justify the dimensions adopted in this study.
- Example of improvement:
*“Digital literacy has been defined from technical (Ontiveros et al., 2006), critical (Gutiérrez, 2003), and socio-cognitive perspectives (Raharjo et al., 2024). This study adopts Torres’ (2023) three dimensions—instrumental, cognitive, and emotional—because they enable a multidimensional analysis of public administration users”.*

Methods:

- Weakness: The selection of the vulnerable population (older adults as a priority group) lacks justification.
- How to improve: Explain why vulnerability supports this choice.
- Example of improvement:
*“Older adults were prioritized in the stratified sample because they represent the group most affected by digital exclusion, offering key insights into access challenges in public digital services”.*

Results:

- Weakness: The section is too descriptive and repetitive, focusing mainly on percentages and tables.
- How to improve: Provide more synthetic narratives directly tied to the hypothesis.
- Example of improvement:
*“Over 62% of respondents achieved a high level of digital literacy, which was associated with more frequent use of public digital services. However, 37% remained at an intermediate level, highlighting the need for specific training programs”.*

Discussion:

- Weakness: Too many international comparisons, with insufficient emphasis on the local context.
- How to improve: Emphasize how findings address the Lambayeque case and complement them with regional evidence.
- Example of improvement (with Espina, 2025):
*“Unlike findings in South Korea, where digital preparedness was moderate (Hyunseung et al., 2024), the Lambayeque case shows higher readiness but persistent gaps in connectivity, requiring infrastructure investment and localized training programs. These findings align with regional evidence, such as Espina (2025), who identified persistent gaps in the digital transformation of public health services in Latin America, reinforcing the need for policies that prioritize vulnerable populations in digital inclusion strategies. This connection shows that, although the contexts differ—administrative services in Peru and healthcare services in Latin America—both reveal that digital literacy remains a critical factor for equitable access to essential services”. *

Espina (2025) ****[Reference 1]

 Conclusions:

- Weakness: Results are restated without highlighting originality.
- How to improve: Emphasize the novel contribution: digital literacy as a bridge between vulnerable citizens and public administration.
- Example of improvement:
*“The study contributes by showing that digital literacy directly empowers vulnerable populations in Lambayeque to interact more effectively with public services, reducing digital exclusion and fostering civic participation”.*

By addressing the weaknesses identified in each section and integrating the suggested reference to Espina (2025), the study gains greater contextual depth and regional relevance. This addition strengthens the discussion by showing that digital literacy not only improves citizen interaction with public administration in Lambayeque but also connects with broader challenges across Latin America. Together, these improvements position the study as a stronger contribution to policy design and academic debate on digital inclusion and public service delivery.

Is the work clearly and accurately presented and does it cite the current literature?

Yes

If applicable, is the statistical analysis and its interpretation appropriate?

I cannot comment. A qualified statistician is required.

Are all the source data underlying the results available to ensure full reproducibility?

Yes

Is the study design appropriate and is the work technically sound?

Yes

Are the conclusions drawn adequately supported by the results?

Yes

Are sufficient details of methods and analysis provided to allow replication by others?

Yes

Reviewer Expertise:

Lorena Espina-Romero’s research focuses on the intersection of digital transformation, organizational culture, and sustainability, with a strong emphasis on applied studies in Latin America. Her main lines of inquiry include:Digital Transformation and Digital Competencies – Exploring the role of digital skills in shaping organizational culture, human resource management, change management, and business sustainability, particularly in SMEs.Artificial Intelligence in Manufacturing – Investigating challenges and opportunities of AI in industrial processes, innovation ecosystems, and decision-making, using bibliometric analyses to map global trends.Organizational Culture, Sustainability, and Circular Economy – Studying the relationship between circular business models, organizational climate, and dynamic capabilities as drivers of sustainable performance.Higher Education and Digital Pedagogy – Analyzing AI-assisted teaching, doctoral-level scientific writing, and the integration of digital tools for research and education.Neuromarketing and Consumer Behavior – Examining the impact of neuromarketing strategies in social networks and the cultural industries, including the promotion of Latin music in the United States.Knowledge Management and Talent Development – Assessing knowledge acquisition and transfer as key factors in sustainable business performance, along with AI-enhanced strategic talent management.Bibliometrics and Scientific Trends – Conducting bibliometric studies on AI, digital transformation, organizational culture, sustainability, and education, applying advanced mapping techniques to identify knowledge gaps and emerging themes.

I confirm that I have read this submission and believe that I have an appropriate level of expertise to confirm that it is of an acceptable scientific standard, however I have significant reservations, as outlined above.

## References

[1] : Gaps in the Digital Transformation of Public Health in Latin America. *Ceniiac* .2025;1: 10.64923/ceniiac.e0004 10.64923/ceniiac.e0004
